# Quantifying uncertainty in measurement of mercury in suspended particulate matter by cold vapor technique using atomic absorption spectrometry with hydride generator

**DOI:** 10.1186/2193-1801-2-453

**Published:** 2013-09-11

**Authors:** Nahar Singh, Tarushee Ahuja, Vijay Narain Ojha, Daya Soni, S Swarupa Tripathy, Ivo Leito

**Affiliations:** CSIR-National Physical Laboratory, Dr K.S. Krishnan Marg, New Delhi, 110012 India; Department of Applied Chemistry, Delhi College of Engineering, University of Delhi, Bawana Road, Delhi, 110042 India; Institute of Chemistry, University of Tartu, Ravila 14a, Tartu, 50411 Estonia

**Keywords:** Mercury, Atomic absorption spectrometer-hydride generator, Uncertainty evaluation, High volume sampler

## Abstract

As a result of rapid industrialization several chemical forms of organic and inorganic mercury are constantly introduced to the environment and affect humans and animals directly. All forms of mercury have toxic effects; therefore accurate measurement of mercury is of prime importance especially in suspended particulate matter (SPM) collected through high volume sampler (HVS). In the quantification of mercury in SPM samples several steps are involved from sampling to final result. The quality, reliability and confidence level of the analyzed data depends upon the measurement uncertainty of the whole process. Evaluation of measurement uncertainty of results is one of the requirements of the standard ISO/IEC 17025:2005 (European Standard EN IS/ISO/IEC 17025:2005, issue1:1-28, 2006). In the presented study the uncertainty estimation in mercury determination in suspended particulate matter (SPM) has been carried out using cold vapor Atomic Absorption Spectrometer-Hydride Generator (AAS-HG) technique followed by wet chemical digestion process. For the calculation of uncertainty, we have considered many general potential sources of uncertainty. After the analysis of data of seven diverse sites of Delhi, it has been concluded that the mercury concentration varies from 1.59 ± 0.37 to 14.5 ± 2.9 ng/m^3^ with 95% confidence level (*k* = 2).

## Introduction

Mercury is a highly toxic element found as naturally and as a contaminant in the environment. Natural sources of atmospheric mercury include volcanoes, geologic deposits, and volatilization from the ocean, rocks, sediments, water, and soils. The toxic effects of mercury depend on its chemical form and the route of exposure. Methyl mercury [CH_3_Hg^+^] is the most toxic form, absorbed readily and excreted slower than other forms of mercury. It affects the immune system, damages the nervous system, alters genetic enzyme systems and also affects the senses of touch, taste, and sight.

There are several instrumental techniques like Atomic Fluorescence Spectrometry (AFS), inductively coupled plasma atomic emission spectroscopy (ICPAES), nevertheless, the cold vapor AAS is the best technique sensitivity wise and to take care of interference of the other species during measurements. Several researchers (Singh and Sarkar 2003; Walcerz et al. 1993; Erdem and Henden 2004 and Barth et al. 1992animals and human beings has been studied by Moore and Ramamoorthy 1984 and indicated the natural and anthropogenic sources through which it reaches in different medium. Laser ionization mass spectrometric investigations have shown that stratosphere contains 1–5 ng m^-3^ of mercury and out of which 90% mercury is present as gas-phase (Murphy et al. 1998; Schroeder and Munthe 1998) as elemental mercury. The concentrations of gaseous mercury and other trace metals in aerosols were measured at Hel site, Gulf of Gdansk (Bełdowska et al. 2006) during summer season. Elemental mercury vapor released from broken thermometers, causes tremors, gingivitis, and excitability when inhaled over a long period of time.

In chemical metrology most of the important decisions are based on the quality and the reliability of analytical results. This reliability, quality and confidence can be achieved by correct estimation of uncertainty of the measurements following the ISO/EURACHEM guidelines. In this regard ISO has published, “Guide to the expression of uncertainty in measurement (GUM)” in 1995 in collaboration with BIPM, IEC, IFCC, IUPAC, and OIML, which recently was revised as VIM: JCGM 200:^2008^, Evaluation of measurement data-Guide to the expression of uncertainty in measurement. In last several years the uncertainty estimations for Hg in different matrixes (Kayal et al. 2009; & Kayal and Singh 2010 Synek et al. 2000; Kumar and Riyazuddin, 2007) have been carried out by various authors. However, determination of mercury in suspended particulate matter (SPM) and evaluating the uncertainty value of the result is a difficult task as there are many parameters involved from sampling to final value, which influence the result. To the best of our knowledge there is no study published, which describes the determinations of mercury in SPM samples with the total uncertainty budget. So in this study, an attempt has been made for the evaluation and expression of uncertainty in measurement for the determination of mercury in environmental samples collected through HVS by AAS-HG, following the ISO GUM guideline (JCGM 100:^2008^) as well as additional relevant guidance documents (ISO ISO/DTS 21748:^2003^ and European Standard EN ISO/IEC 17025:^2005^).

### Experimental details

#### Instruments and apparatus

The suspended particulate matter (SPM) sampling was carried out using a High-Volume Sampler (HVS-410, Make: Envirotech). SPM was collected on pre-desiccated cellulose filter papers. Before sampling, the filter papers were dried for 3 hrs in an oven at 105°C to remove moisture, if any, and to get constant weight. After drying, the filter papers were kept in a desiccator at room temperature. For weighing of sample Mettler Toledo make balance model AX 204 capacity 220 g has been used. Atomic Absorption Spectrometer (AAS) of Analytik Jena make Vario-6 with a hydride generator accessory was used for the analysis of mercury. The mercury measurements were carried out at 253.7 nm wavelength at optimum conditions of acids and reducing agent. The pipette of 5 mL and volumetric flask of 50 mL capacity used were of Borosil glass works, India Limited. The pipettes and volumetric flasks were calibrated prior to analysis by the National Physical Laboratory (National Metrology Institute of India) following international standard procedure and protocol. The reference standards stock solution of 1.0 mg/litre (BND 601.02) of mercury was used (prepared by NPL-India) after diluting up to the working range for the calibration of AAS-HG instrument. A reagent blank was also prepared and the correction applied wherever required. Mean absorbance value of the three measurements of two replicates of each sample was taken into consideration for the calculation of the concentration of mercury. All the acid digestion and dilution work was carried out in a cleaned laminar flow bench equipped with the proper exhaustive system.

#### Reagents

Hydrochloric acid (35%) and Nitric acid (69%), of GR grade (Guaranteed Reagent), which were further purified by sub boiling point distillation in a quartz glass device. Hydrogen peroxide (50%) and Sulphuric acid (98%) all of E. Merck (India) make were used. De-ionized water (18.2 MΩ resistivity) prepared from Millipore milli-Q element water purification system, USA was used throughout the process.

#### Sampling sites and description

New Delhi is located 160 km south of the Himalayas at latitude 28°24’ to 28°53’N and longitude 76°20’E to 77°20’E with an altitude of about 216 meter above mean sea level. Delhi’s climate is mainly influenced by its inland position and the prevalence of continental air during major part of the year. Delhi has three distinct seasons: summer, monsoon and winter. In the proposed study seven sites were selected namely Ashram (S-1; Heavy traffic site), Azadpur (S-2; Industrial site), Loni Road (S-3; densely populated + traffic site), Pitampura (S-4; Ambient site), Highway no. 56 (S-5; Traffic site), Naraina (S-6; Industrial site) and NPL (S-7; Mixed site).

### Sample preparation procedure

Two replicates of 18 × 11.5 cm^2^ size (known weight at deposited area) of all the seven sites were taken for the determination of mercury into separately cleaned poly-tetrafluoroethylene (PTFE) vessels used in the microwave digestion system. To these vessels 15 mL of sub boiled nitric acid, 5 mL of hydrogen peroxide and 5 mL of DI water were added and closed tightly with PTFE vessel lid. The PTFE vessels containing the samples were kept for overnight at room temperature. After keeping the PTFE vessel overnight, 4 to 5 drops of sulphuric acid was added and the PTFE vessel was kept on hot plate by covering with Teflon lid at 100°C for 15–20 minutes and then heated again at 150°C. Then the lid of the PTFE vessel was opened and it was heated again after addition of 10 ml sub boiled concentrated hydrochloric acid. To ensure the complete removal of nitric acid the sample was heated once again with 5 mL hydrochloric acid. Finally the solutions were boiled with 15 ml 1:1 de-ionized water and sub boiled concentrated hydrochloric acid mixture. After digestion the whole content of the vessel was transferred into a 100 mL quartz centrifuge tube and it was centrifuged at 5000 rotations per minute for 2–3 minutes to separate solid particles from the solutions. The supernatant liquid was transferred into a glass beaker. The remaining residue in the vessel was washed several times with hot water to ensure complete transfer of digested sample from the vessel. The final solutions were made 50 mL by addition of de-ionized water. In this solution mercury was determined by AAS-HG using the respective standards and principal resonance line after reducing mercury in the presence of sodium borohydride (3%), sodium hydroxide (1%) and hydrochloric acid (1%) by cold vapor techniques (ambient room temperature). Mean absorbance values of six readings of two replicates (three each) were taken into the consideration for calculation of the concentration of mercury. Procedural blank for cellulose filter paper without sample was also done to check the blank levels for mercury and correction was applied wherever required.

### Determination of recovery

For determining the recovery of the wet digestion procedure we have taken three separate SPM deposit filter papers of the same matrix assuming uniform deposition of mercury on the filter paper. The filter paper was divided in two parts (18 cm × 11.5 cm^2^ each). One part was treated as such, while in the second part 10 ml of 1000 μg/L mercury solution was added in a PTFE vessel along with the filter paper and the remaining procedure was the same as in the case of test samples. The dilutions of the spiked samples were carried out in the calibration range of AAS-HG before analysis. When keeping samples for long time there is a danger of loss of mercury in the low concentration range. In the present study we analyzed all samples within one week after digestion. From our experience we know that when we analyze mercury by AAS-HG in the low concentration range, we have to prepare fresh standards every week (calibration range: 10 μg/L to 50 in μg/L). After one week, there is a loss of 5% to 7% in the predetermined value at 27 ± 3°C (ambient conditions of our lab). The concentration range of mercury in the samples was 15 μg/L to 30 μg/L. The concentration of hydrochloric acid in the final solution should be at least 4-5%. In 4% or higher concentration of hydrochloric acid there is no loss due to adsorption over glass substrate, however mercury may be lost due to evaporation and microbial growth within the solution.

## Determination of concentration of mercury in SPM sample and its uncertainty

### Measurement model

In the determination of mercury in SPM samples the major sources of the uncertainty have been included in the calculation of combined uncertainty according to the EURACHEM/GUM guidelines. Following EURACHEM/GUM guidelines concentration of mercury *C(M*_*Hg*_*)* in the SPM samples has been evaluated using AAS-HG technique by following equation.1

Where; C(*M*_*Hg*_) = Concentration of mercury in ng/m^3^; C_*Hg*_ = Concentration of mercury analyzed from AAS-HG in μg/litre; V_*TEST*_ = Volume made of the test sample after wet digestion process in mL; *W*_*TSPM*_ = Total weight of SPM deposit on 18 cm × 23 cm area; V_*HVS*_ = volume of the air processed through high volume sampler (HVS); W_*ANA*_ = Weight of SPM deposit on 18 cm × 11.5 cm area taken for wet digestion process. R_*HVS*_ = Total recovery of the method containing the contribution of HVS in capturing mercury and possible losses of mercury during sample preparation.

**Figure 1:**
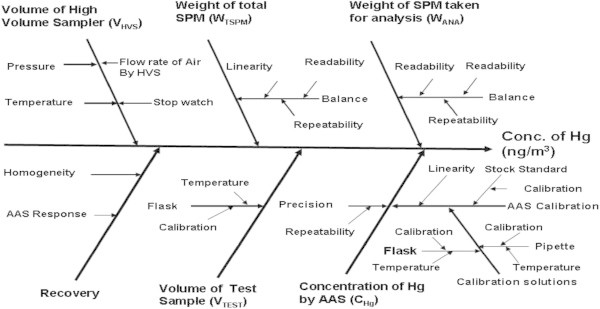
**Fish bone diagram for potential sources of uncertainty in mercury measurement by HG-AAS.**

The evaluated values for the above factors are given in Table  1. The important parameters that contribute towards the uncertainty are also shown in the Cause and effect diagram in Figure 1. In accordance with GUM, the combined uncertainty for the mathematical model, which is in a product or quotient form, is given by:2

**Table 1:** **Various evaluated components for the determination of mercury in SPM samples**

Sample I.D.	SPM (μg/m^3^)	Concentration of mercury obtained from AAS-HG after reducing blank (μg/litre) [C_Hg_]	Volume made for test samples (mL) [***V***_***TEST***_]	Total weight of SPM deposited on 18 cm × 23 cm filter paper (g) [***W***_***TSPM***_]	Weight of SPM deposited on 18 cm × 11.5 cm filter paper area taken for analysis (g) [***W***_***ANA***_]	Volume of air processed through HVS at STP, (m^3^) [***V***_***HVS***_]	Recovery of the analytical procedure (%) [***R***_HVS_]	**Concentration of mercury in ng/m^3^C(M_Hg_)
S-1	395	58.22 (2.82)*	50	0.17744	0.08872	493.52	94.39	12.55 ± 2.44
S-2	203	23.41 (2.13)*	50	0.09124	0.04562	414.97		6.00 ± 1.23
S-3	2337	62.32 (4.13)*	50	1.05086	0.52543	457.16		14.50 ± 2.87
S-4	251	6.83 (1.12)*	50	0.11279	0.05639	456.66		1.59 ± 0.37
S-5	338	14.04 (0.92)*	50	0.15214	0.07607	471.24		3.17 ± 0.63
S-6	429	23.11 (1.62)*	50	0.19300	0.09650	417.58		5.89 ± 1.17
S-7	347	16.6 (1.33)*	50	0.62306	0.31153	391.63		4.51 ± 0.91

*Value in parentheses is the standard deviation of six determinations of two replicates (three each).

**Concentration of metal mercury has been calculated by using eq.-1 and values reported with expanded uncertainty in ng/m^3^.

The combined uncertainty is given by:3

The uncertainty evaluation of all the input quantities is discussed in the following sub-sections.

### Practicalities of uncertainty estimation

An uncertainty source may be ‘Type A’, which is evaluated by statistical analysis of a series of observations, or ‘Type B’, which is evaluated by using means other than the statistical analysis of a series of observations. To calculate standard uncertainty from the parameters of two most important distribution functions, if the limits x ± a are given without a confidence limit, it is appropriate to assume a rectangular distribution with a standard deviation of a/√3, but if values are given with confidence level, it is triangular distribution with a standard deviation of a/√6 (Ellison et al. 2000). Normal distribution is assumed when an estimate is made from repeated observation of a randomly varying process. In analytical chemistry, an expanded uncertainty (U) is used, which is obtained by multiplying the combined standard uncertainty, by a coverage factor k. The choice of coverage factor is based on the level of confidence. For an approximate level of confidence of 95%, the value of k is 2.

### Uncertainty components in the measurement of concentration of mercury obtained from AAS-HG [*C*_*Hg*_]

Referring to Figure 1, the major sources of the uncertainty for C_*Hg*_ are due to reference standard stock solution, repeatability, dilution in 50 mL volumetric flask from stock solution to working range and five mL capacity pipette used for dilution.

### Reference standard stock solution

According to the certificate the concentration of the mercury standard stock solution is 1.00 ± 0.02 mg/L. Assuming normal distribution the standard uncertainty will be 0.01 mg/L. This stock solution was used for calibration of AAS-HG after proper dilution up to the working range.

### Uncertainty evaluation due to repeatability

The repeatability in concentration of mercury was determined experimentally by analyzing six determinations of two independently digested replicates (three each). The standard deviation of six values (three each) has been used for calculation of standard uncertainty following EURACHEM guide.

### Uncertainty in 50 ml volumetric flask used for dilution from 1000 ug/L to working range

The final volume of standard mercury solution made was 50 mL after several dilutions. The factors which include uncertainty in 50 ml volumetric flask are given in Figure 1. The major uncertainties were due to calibration and temperature. The uncertainty due to calibration and temperature has been calculated as below.

### Uncertainty due to calibration

As per calibration certificate of supplier the expanded uncertainty for 50 mL flask is ± 0.02 mL at k = 2 assuming normal distribution, hence the standard uncertainty will be u(V_*cal*_) = 0.01 mL.

### Uncertainty due to temperature

According to calibration certificate the flask has been calibrated at 25°C and the laboratory temperature varies within the limits of ± 3°C. The volume expansion coefficient for water is 2.1 × 10^-4^°C^-1^, which leads to volume variation according to following equation.4

Where V is the volume of volumetric flask and ΔT is temperature variation in the laboratory. Volume variation for 50 mL flask = ± (50 × 3 × 2.1 × 10^− 4^) = 0.0315 *mL*.

The standard uncertainty due to temperature effect, u(V_T_) is calculated using assumption of a rectangular distribution i.e. u(*V*_T_) = 0.0315/√3 = 0.018 mL.

The combined uncertainty for temperature and calibration in 50 mL volumetric flask has been calculated as; 

### Uncertainty due to five mL pipette used for dilution

5 mL aliquot was taken by 5 mL pipette for making dilution upto 50 mL. As per Figure 1 the main uncertainties were due to temperature variation and calibration accuracy (± 0.02 mL) assuming normal distribution as quoted by the manufacturer. The effect of temperature on 5 mL pipette has also been determined as per section, uncertainty in 50 ml volumetric flask used for dilution from 1000 ug/L to working range. The uncertainty of every dilution is different but this difference is negligible. Therefore for simplicity we have considered the uncertainty of first dilution even after performing the dilution second times.

The Combined uncertainty due to measurement of concentration of mercury was calculated using data of subsection; uncertainty in reference standard stock solution, Uncertainty evaluation due to repeatability, Uncertainty in 50 ml volumetric flask used for dilution from 1000 ug/L to working range and Uncertainty due to five mL pipette used for dilution in following equation.5

### Uncertainty due to recovery

The recovery of mercury from the SPM filter paper depends on the storage of the samples and the method adopted for digestion. The loss of mercury in a closed system is less problematic during digestion process in comparison to an open system. The loss of mercury while keeping at low temperature i.e. 10 ± 5°C has been found at our laboratory to be 15-20%, while at ambient temperature 30 ± 5°C the loss of mercury was around 45–50% (both during one year period). For determining recovery of the wet digestion procedure we have taken three separate SPM deposit filter papers with the same matrix assuming uniform deposition of mercury on the filter paper. The mercury deposited filter paper was divided in two parts (18 cm × 11.5 cm each). One part was treated as such, while in the second part (200 μg/L) 10 ml of 1000 μg/L were added in the PTFE vessel along with filter paper and the remaining procedure was the same as in the case of test samples. The final solutions after digestion were made to 50 mL by de-ionized water. The dilutions of the spiked samples were carried out in the calibration range of AAS-HG before analysis. The recovery has been done as; Recovery % = Concentration of spiked sample/ (200 μg/L + concentration of non spiked sample). Following the same approach the average recovery of the spiked samples were found to be 94.39 ± 0.80%.

### Uncertainty evaluation in 50 mL volumetric flask used in making volume after wet chemical digestion [V_*TEST*_]

The known area having known weight of the sample was processed through wet chemical route and the final volume was made to 50 mL. Referring to Figure 1, the sources of uncertainty for V_*TEST*_ are due to calibration uncertainty and effect of temperature variation on 50 mL volumetric flask volume of aliquot taken. The uncertainty associated with temperature for 50 mL volumetric flask is carried out as per section 5.3. The combined uncertainty for V_*TEST*_ has been calculated on the basis of Figure 1 as follows;

### Uncertainty evaluation due to volume of air processed through high volume sampler [V_*HVS*_]

The volume of air sampled through HVS has been calculated on the basis of equation-6 and the volume of air processed at standard temperature and pressure (STP) is calculated by using equation-7 given below.67

Where, V_1_- Total volume of air processed through HVS, P_1_- Atmospheric pressure on the date of measurement (mm of mercury); T_1_- Standard temperature (273); T_2_- working temperature [temperature of the sampling site °C + standard temperature] + standard temperature] and P_2_- Standard pressure (760 mm of mercury).

Substituting value from Table  2 in eq.7

**Table 2:** **Uncertainty due to high volume sampler (*V***_***HVS***_**) for measuring volume of air at STP**

Name of the site	Date of sampling	Sampling starting time	Sampling end time	Flow rate of air sampled through HVS (m^3^/min)	Time (in min.)	Volume of air processed through HVS (m^3^) V_1_	Atmospheric pressure measured on site (mbar)	Atmospheric pressure in mm of mercury (mbar × 0.75) P_1_	Average temperature of the sampling site (°C)	Average temperature of the sampling site (K) T_1_	Volume of air processed through HVS at STP (m^3^) [***V***_***HVS***_]*
**Heavy traffic site; Ashram zone (S-1)**	31-1-08	9.10 am	5.30 pm	1.1	500	550	994.2	745.65	25.5	298.5	493.52
**Industrial site; Azadpur zone (S-2);**	1-2-08	10.0 am	5.00 pm	1.1	420	462	995.2	746.40	25.5	298.5	414.97
**Densely populated + Traffic site Loni Road (S-3);**	2-2-08	9.45 am	5.30 pm	1.1	465	511.5	993.6	745.20	26.5	299.5	457.16
**Ambient site Pitampura zone (S-4);**	3-2-08	9.45 am	5.30 pm	1.1	465	511.5	992.5	744.37	26.5	299.5	456.66
**Traffic zone; Highway No. 56 (S-5);**	4-2-08	10.30 am	6.30 pm	1.1	480	528	995.5	746.63	27.5	300.5	471.24
**Industrial site; Nariana zone (S-6)**	5-2-08	9.15 am	6.00 pm	1.1	425	467.5	996.3	747.22	27.5	300.5	417.58
**Mixed site; NPL zone (S-7)**	6-2-08	10.0 am	4.40 pm	1.1	400	440	992.8	744.60	27.5	300.5	391.63

*[V_*HVS*_] is calculated using equation -7, following section uncertainty evaluation due to volume of air processed through high Volume sampler in section- 8.

Similarly V_*HVS*_ for sample S-2 to S-7 has been calculated. Referring to Figure 1 the sources of the uncertainty for V_*HVS*_ are flow rate of air through HVS, time recording by stop watch, ambient pressure measurement and measurement of temperature of the site. The uncertainty estimates of the parameters are taken from the calibration certificates. The sampling is carried out in open atmosphere so it is not possible to control the temperature of the atmosphere. So we have taken normal distribution for temperature variation, while the other analytical experiments were carried at controlled room temperature. The combined standard uncertainty for volume of HVS has been calculated using equation-8.8

### Uncertainty evaluation due to balance used for weighing the blank and SPM deposited filter paper [*W*_*TSPM*_] and [*W*_*ANA*_]

As per the certificate of balance the Linearity (mg) /readability (mg) / repeatability (mg); ± 0.2/0.01/0.07 has been used for calculation and the standard uncertainty has been calculated assuming rectangular distribution and dividing each by √3. The contribution needs to be counted twice, once for tare, and the other for gross weight. So the combined standard uncertainty due to balance is 0.00024 g.

The total weight of the SPM deposited on filter paper and weight of the filter paper used for wet digestion process was calculated as follows;

(I) Total area of the cellulose filter paper for sampling =20.3 cm × 25.4 cm = 515.62 cm^2^
(II) Total area of the filter paper used for deposition (18 cm × 23 cm) = 414 cm^2^
(III) Total weight of the cellulose filter paper used for sampling (515.6 cm^2^) = 4.25504 g
(IV) Total weight of the cellulose filter paper after deposition (515.6 cm^2^) = 4.47603 g
(V) Total suspended particulate matter deposit on the filter paper (515.6 cm^2^) = (IV-III) = 0.22099 g
(VI) Total suspended particulate matter deposit on the filter paper (*414 cm^2^); W_TSPM =_
* It has been considered that the thickness of the cellulose filter used is uniform throughout the area. On this basis we have calculated the weight of the deposit area, which is 414 cm^2^.
(VII) Weight of the SPM deposited area (18 cm × 11.5 cm) taken for analysis, W_*ANA*_ = 0.08872 g. The weight calculations for samples S-2 to S-7 were also done in same way as given above for sample-1.

### Calculation of concentration and associated uncertainty of mercury for S-1; (ng/m^3^)

The concentration of mercury in the seven SPM samples has been determined by AAS-HG using eq.1. Substituting the values from Table  1 into eq.1, the concentration of mercury (ng/m^3^) in S-1 sample: .

The combined uncertainty in measurement of mercury have been calculated after putting values of various parameters from Table  3 (summary table) in eq.3.

**Table 3:** **Uncertainty components (summary table) for calculation of combined uncertainty**

S.N.	u(C_***Hg***_)/ C_***Hg***_	u(V_***TEST***_)/ V_***TEST***_	u(W_***TSPM***_)/ W_***TSPM***_	u(W_***ANA***_)/ W_***ANA***_	u(V_***HVS***_)/ V_***HVS***_	u(R)/R
S-1	1.30/ 58.22	0.021/50	0.00024/ 0.17744	0.00024/ 0.08872	46.54/ 493.52	0.80/ 94.39
S-2	0.90/ 23.41	0.021/50	0.00024/ 0.09124	0.00024/ 0.04562	39.13/ 414.97	0.80/ 94.39
S-3	1.80/ 62.32	0.021/50	0.00024/ 1.05086	0.00024/ 0.52543	43.11/ 457.16	0.80/ 94.39
S-4	0.46/ 6.83	0.021/50	0.00024/ 0.11279	0.00024/ 0.05639	43.06/ 456.66	0.80/ 94.39
S-5	0.40/ 14.04	0.021/50	0.00024/ 0.15214	0.00024/ 0.07607	44.44/ 471.24	0.80/ 94.39
S-6	0.70/ 23.11	0.021/50	0.00024/ 0.19300	0.00024/ 0.09650	39.38/ 417.58	0.80/ 94.39
S-7	0.57/ 16.6	0.021/50	0.00024/ 0.62306	0.00024/ 0.31153	36.93/ 391.63	0.80/ 94.39

**Figure 2:**
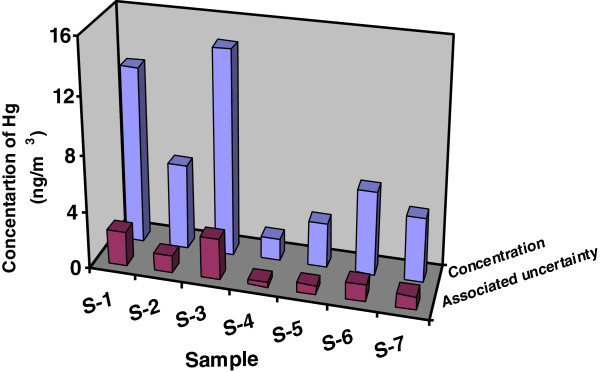
**Concentration of each sample with associated uncertainty value.**

Therefore, Concentration of mercury in sample S-1 is = 12.5 ± 2.4 ng/m^3^. Similarly concentration of mercury and combined uncertainty for mercury measurements have been calculated for samples S-2 to S-7. Table  4 shows the analytical results of the seven sites and a graphical representation of mercury concentration along with associated uncertainty is given in Figure 2.

**Table 4:** **Results of mercury with uncertainty in SPM analyzed by AAS-HG**

Name of the site	SPM in μg/m^3^	Concentration of mercury in ng/m^3^with expanded uncertainty at 95% confidence; k = 2
Heavy traffic site (S-1); Ashram zone	395	12.6 ± 2.4
Industrial site (S-2) ; Azadpur zone	203	6.0 ± 1.2
Densely populated + Traffic site (S-3); Loni Road	2,337	14.5 ± 2.9
Ambient site (S-4) ; Pitampura zone	251	1.59 ± 0.37
Traffic site (S-5); Highway No.56	338	3.17 ± 0.63
Industrial site (S-6) ; Nariana zone	429	5.9 ± 1.2
Mix Zone (S-7); NPL zone	347	4.51 ± 0.91

## Result and discussion

Accurate determination of mercury in air is not easy due to the difficulties in measuring the air volume, losses during sample digestion process due to its vaporizing nature. The evaluation of combined uncertainty in mercury concentration in SPM sample is very complicated as there are various parameters, which contribute uncertainty in the entire process. On determining the uncertainty for mercury content measurement we were redundantly meticulous in identifying all uncertainty sources. In a routine determination of uncertainty we recommended that it is possible to be much less pedantic, and only reveal and realistically quantify the most significant components. The uncertainty components, which contribute a negligible value, could be ignored from the calculation of combined uncertainty value. The uncertainty components, which are found to contribute significantly during the subsequent computations, can be quantified more precisely and the final combined uncertainty can be recalculated. Mae and Dan 2010 also suggested how uncertainty can be reduced in measurement of atmospheric mercury and also suggested mercury in the air is measured as three forms like gaseous elemental mercury, reactive gaseous or gaseous oxidized and particle-bound mercury.

The concentration of mercury on the S-4 site is 1.57 ± 0.37 ng/m^3^, which is the lowest (ambient site), while on the S-4 site (traffic/densely populated) the gighest concentration 14.5 ± 2.9 ng/m^3^ was observed. The relative uncertainty in the measurement of mercury by FAAS-HG varies from 19 to 23%. Gajghate et al. 2011 have also estimated analytical uncertainty in quantification of mercury in different fractions of PM_10_ using microwave digestion and DMA-80 mercury analyzer. The mercury concentrations in ambient air in different various fractions of PM_10_ were found to be in the range of 3.13 ng m^-3^ (26%) – 22.96 ng m^-3^ (15%) with respective expanded relative uncertainty. Synek et al. 2000 estimated uncertainties in mercury determinations in biological materials by atomic absorption spectrometer – AMA 254 and suggested major uncertainty (52%) is due to sample absorbance measurement.

**Figure 3:**
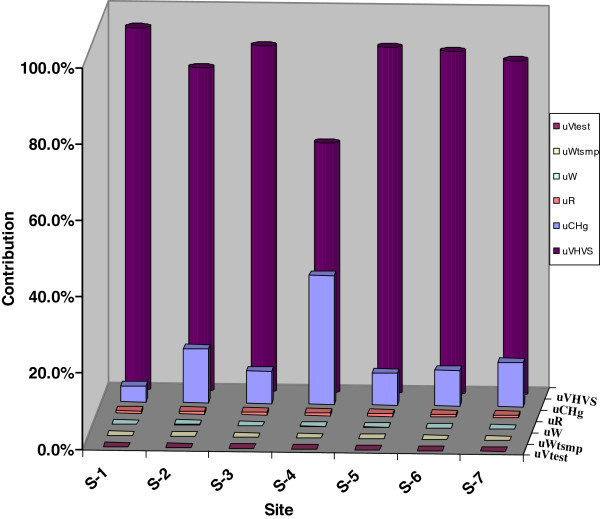
**Contribution of each uncertainty source.**

In the proposed determination of mercury concentration (S-1), there are five main uncertainty sources, which directly influence the final results those are *C*_*Hg*_, *V*_*TEST*_*, W*_*TSPM*_*, W*_*ANA,*_*V*_*HVS*__*and*_*R*_*HVS*_, with relative contributions 4.2%, 0.001%, 0.02%, 0.06%, 95.1% and 0.6% respectively. It is clear from the Figure 3 that flow rate (*V*_*HVS*_) is the crucial parameter, which contributes the highest uncertainty of the value. The flow rate can be changed by several factors such as oily nature of the sample, presence of photochemical smog; wood smoke. These factors may block the filter paper and cause a rapid drop in air flow. Besides these high humidity and dense fog can cause the filter to become wet and thus airflow reduces through the filter paper. So sampling through HVS is the additional source, which also contributes uncertainty in mercury determination. Traffic and industry are the major sources, which contribute major contamination of mercury in environment.
